# Gene Expression Profiling of Biological Pathway Alterations by Radiation Exposure 

**DOI:** 10.1155/2014/834087

**Published:** 2014-09-08

**Authors:** Kuei-Fang Lee, Julia Tzu-Ya Weng, Paul Wei-Che Hsu, Yu-Hsiang Chi, Ching-Kai Chen, Ingrid Y. Liu, Yi-Cheng Chen, Lawrence Shih-Hsin Wu

**Affiliations:** ^1^Institute of Medical Sciences, Tzu Chi University, Hualien 970, Taiwan; ^2^Laboratory for Cytogenetics, Center for Genetic Counseling, Buddhist Tzu Chi General Hospital, Hualien 970, Taiwan; ^3^Innovation Center for Big Data and Digital Convergence, Yuan Ze University, Chungli 32003, Taiwan; ^4^Department of Computer Science and Engineering, Yuan Ze University, Chungli 32003, Taiwan; ^5^Bioinformatics Core Laboratory, Institute of Molecular Biology, Academia Sinica, Taipei 11529, Taiwan; ^6^Department of Molecular Biology and Human Genetics, Tzu Chi University, Hualien 970, Taiwan; ^7^Department of Computer Science & Information Engineering, Tamkang University, New Taipei City 25137, Taiwan

## Abstract

Though damage caused by radiation has been the focus of rigorous research, the mechanisms through which radiation exerts harmful effects on cells are complex and not well-understood. In particular, the influence of low dose radiation exposure on the regulation of genes and pathways remains unclear. In an attempt to investigate the molecular alterations induced by varying doses of radiation, a genome-wide expression analysis was conducted. Peripheral blood mononuclear cells were collected from five participants and each sample was subjected to 0.5 Gy, 1 Gy, 2.5 Gy, and 5 Gy of cobalt 60 radiation, followed by array-based expression profiling. Gene set enrichment analysis indicated that the immune system and cancer development pathways appeared to be the major affected targets by radiation exposure. Therefore, 1 Gy radioactive exposure seemed to be a critical threshold dosage. In fact, after 1 Gy radiation exposure, expression levels of several genes including FADD, TNFRSF10B, TNFRSF8, TNFRSF10A, TNFSF10, TNFSF8, CASP1, and CASP4 that are associated with carcinogenesis and metabolic disorders showed significant alterations. Our results suggest that exposure to low-dose radiation may elicit changes in metabolic and immune pathways, potentially increasing the risk of immune dysfunctions and metabolic disorders.

## 1. Introduction

In contrast to the hazards of high-dose radiation exposure, the damage effects of low-dose radiation are not well-understood yet [1, 2]. Such information is necessary in order of establish regulatory procedures of radiation protection. The United Nations Scientific Committee on the Effects of Atomic Radiation (UNSCEAR) defined 200 mSv as low, 200–2000 mSv as medium, and over 2000 mSv as high dose [3]. No longitudinal epidemiological investigation has yet been performed and no direct evidences of damage induced by low-dose radiation exposure have been found [4]. Therefore, the International Commission on Radiological Protection (ICRP) for radiation safety made a conservative assumption: the linear, nonthreshold (LNT) hypothesis—any long-term, biological damage caused by ionizing radiation (usually cancer) is directly correlated with the radiation exposure, without taking into consideration the possibility that variations in radiation dosages may generate different effects [5–7]. As such, all radiation dosages, whether high or low, are always considered harmful. There are essentially no well-established safety thresholds.

Existing radiation injury studies have primarily focused on changes in the immune system and progression towards carcinogenesis as a result of radiation exposure. Immune system responses can be influenced by variations in the genes and environment, which includes biological factors like pathogens and external factors such as ionizing radiation [8, 9]. High-dose radiation (>1 Gy) has been shown to severely disrupt immune system functions, leading to a drastic increase in the death of blood cells in mice [10, 11]. Moreover, in patients with acute radiation syndrome, hematopoietic cell proliferation was found to be inhibited by radiation exposure [12]. These findings indicate that high-dose radiation has a destructive effect on the immune system. However, the extent to which radiation dose <1 Gy can affect the immune system is still not clear. Previous studies have shown that damage to the hematopoietic system and immune system is reduced at low ionizing radiation doses [13, 14]. In fact, long-term accumulative radiation dose (<1 Gy) appears to enhance the resilience and tolerance of cells. It was observed that, under such a condition, even though T lymphocyte proliferation was inhibited, the innate immune system and naive T cell differentiation were activated and immune functions were enhanced, while the activity and maturation of dendritic cells were unaffected [9, 15, 16]. These findings also suggest that sensitivity and tolerance to radiation are different among the variety of immune cells in the body.

The association between low-dose radiation and carcinogenesis is controversial. However, some studies have provided evidence to support the ability of low-dose radiation to suppress the aging process, delay cancer progression, enhance immune functions, and promote growth and development [17]. A multistage cancer model was used to describe the putative rate-limiting steps in carcinogenesis in association with radiation hormesis, suggesting a positive impact of radiation therapy on the incidence of lung cancer and the potential benefit of low-dose radiation stimulus on enhancing DNA repair and reducing carcinogenesis risk [18, 19].

Nonetheless, very few existing studies have fully demonstrated the effects of low-dose radiation on a genome-wide scale. Recent gene expression profiling analyses support the use of biomarkers for the estimation of radiation biodosimetry [20, 21]. These studies revealed that genes involved in cellular structural integrity, immune functions, cell cycle control, and apoptosis were more responsive to radiation. In particular, alterations in the expression of genes responsible for the formation and maintenance of cellular structure and cell cycle control may lead to chromosome instability and carcinogenesis [22–25]. Unfortunately, changes in the transcriptome elicited by different radiation dosages have not been fully investigated.

In the present study, we attempted to examine the effects of varying doses of cobalt 60 radiation in human peripheral blood mononuclear cells. By integrating array-based gene expression profiling with subsequent systematic bioinformatics analyses, we uncovered radiation sensitive genes concentrated in certain chromosomal regions, specific gene expression patterns associated with different radiation dosages, and important immune and cancer-related pathways responsive to radiation exposure.

## 2. Material and Methods

### 2.1. Sample Preparation

Blood samples (30 mL per subject) are obtained from five participants and collected into vacutainers containing sodium heparin. Samples were irradiated using ^60^Co at a dose rate of 0.546 Gy/min (The Institute of Nuclear Energy Research (INER), Taoyuan, Taiwan). The radiation doses used in these experiments were chosen to cover a range of doses: 0.5 Gy, 1 Gy, 2.5 Gy, and 5 Gy. The control RNA samples were not exposed to any radiation. Samples were harvested after 24 hours of treatment with radiation [26, 27]. Informed consent was obtained from all participants. All procedures were approved by the Institutional Review Board at Tzu Chi General Hospital, Hualien, Taiwan.

### 2.2. RNA Preparation

Total RNA was isolated from peripheral blood mononuclear cells using Trizol. RNA quality was determined by an OD260/280 ratio ≥1.8 and OD260/230 ratio ≥1.6 on a spectrophotometer and the intensity of the 18S and 28S rRNA bands on a 1% formaldehyde-agarose gel. RNA was detected on a spectrophotometer. RNA integrity was examined on an Agilent 2100 Bioanalyzer (Agilent Technologies, Inc., USA). RNA samples with a RIN (RNA integrity number) of ≥6.0 and 18S/28S >0.7 was subjected to microarray analysis.

### 2.3. Microarray Data Analysis

One microgram of total RNA was prepared for the cDNA reversed transcription reaction and using Amino Allyl MessageAmp II aRNA Amplification Kit (Ambion number AM1753, CA, USA) according to the manufacturer's instructional resources information system. Double stranded cDNA was synthesized and followed by an in vitro transcription reaction to amplify aRNA incorporated with biotin labeling system for the microarray hybridization in triplicate. The Cy5-labeled aRNAs were fragmented by using the reagents and protocol provided in Ambion RNA Fragmentation Reagents kit (Ambion Inc., Austin, TX) for microarray hybridization to the Human Whole Genome One Array Version 6.1 (HOA 6.1, Phalanx Biotech Group, Inc., Taiwan). Nonspecific binding targets were washed out three times.

### 2.4. Statistical Analysis

The arrays were scanned by AXON4000B scanner (Molecular Devices, CA, USA). The fluorescent intensities of each spot were analyzed by GenePix 4 (Molecular Device, CA, USA). The data were averaged from the triplicates and were normalized using Rosetta Resolver System software (Rosetta Biosoftware, USA). Rosetta error models were available for gene expression analysis in five pairwise comparisons. Standard selection criteria to identify differentially expressed genes are as follows: (1) log⁡_2_|Fold  change| ≥ 1 and *P* < 0.05; (2) log⁡_2_ ratios = “NA” and the differences of intensity between the two samples ≥1000. Calculation of reproducibility among the technical replicates was performed by Pearson's correlation coefficient. PCA and clustering analysis were performed on selected differentially expressed gene lists after data transformation and mean centering to cluster genes by averagely linkage algorithm. The correlation of expression profiles between samples and treatment conditions was demonstrated by unsupervised hierarchical clustering analysis.

### 2.5. Bioinformatics Analysis

Differentially expressed genes were used as input for a series of bioinformatics analyses performed with the WEB-based GEne SeT AnaLysis Toolkit (WebGestalt) [28, 29]. WebGestalt is an open analytical platform that integrates gene ontology (GO) [30], KEGG [31], WikiPathway [32], protein interaction networks, microRNA binding sites, and transcription factor targets [MSigDB [33]], as well as cytogenetic band information, for a variety of enrichment analyses. The GO, KEGG, protein interaction network, and cytogenetic band enrichment analytical tools were utilized to analyze the differentially expressed genes. Multiple testing bias was adjusted by a Benjamini-Hochberg threshold of *P* < 0.05, except for the smaller number of differentially expressed genes in the 0.5 Gy dosage group, where a raw *P* value of > 0.01 was applied as the threshold.

## 3. Results

### 3.1. Differentially Expressed Genes (DEGs) Profile Exhibited after Different Exposure Doses of Radiation

Principle component analysis (PCA) was performed to evaluate any differences among biological replicates and their treatment conditions. Data reproducibility was assessed by PCA and clustering analysis shown in Supplemental Figures 1 and 2, available online at http://dx.doi.org/10.1155/2014/834087. The analysis result indicated the experiment of microarray was consistent.

**Figure 1:**
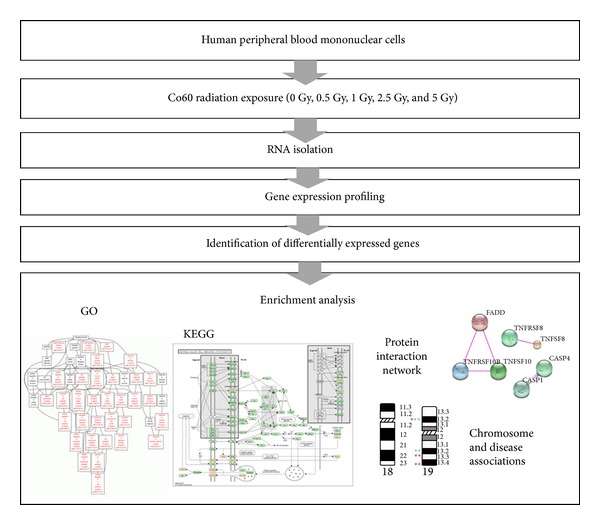
System flow of our analysis.

Table 1 shows the number of significantly differentially expressed genes in human PBMC exposed to varying doses of ^60^Co radiation (absolute Log_2_ ratio ≥1; absolute fold-change ≥2; FDR < 0.05). A radiation dosage of 0.5 Gy did not affect a lot of genes; therefore, this dose probably induces subtle changes in the genome relative to other radiation dosages. In contrast, 1 Gy of radiation dose generated changes in the greatest number of genes; therefore, this must be an important dose. From 1 Gy to 2.5 Gy, there is a drop in the number of genes affected by radiation, but from 2.5 Gy to 5 Gy, there appears to be an opposite trend, suggesting that there are complex regulatory mechanisms underlying these dosage levels (see Figure 1).

### 3.2. Pathway Analysis

Gene ontology enrichment analysis results are given in Table 2 and Supplementary Table 1. It appears that, starting at a dosage of 0.5 Gy  ^60^Co radiation, cells may have already been affected in terms of nucleotide metabolism and signaling pathways responsive to external apoptotic signals. Most of the genes responsive to 1 Gy of  ^60^Co radiation dosage are members of the immune system and programmed cell death. At higher radiation dosages (2.5 Gy and 5 Gy), negative regulation of molecular functions was activated, cell's response to cytokine stimulus was changed, and genes involved in the cytokine-mediated pathway were altered.

Table 3 indicates that different cellular pathways are affected at the various radiation dosages tested in this study. At the radiation dosages (0.5 and 1 Gy), most of the genes affected belong to metabolism and maintenance of regular cell activity in the blood. In contrast, high dosages of radiation significantly altered the genes involved in the MAPK signaling pathway, cell's response to cytotoxicity, and apoptosis.

At 0.5 Gy, the biological processes most affected appeared to be nucleotide metabolism, indicating that radiation dosage may alter basal cellular processes. Beginning at 1 Gy, increasing radiation exposure induces changes in a significant number of genes involved in the immune system processes and programmed cell death pathways. These genes are mostly mapped to chromosomes 2, 11, 16, 17, and 19 (Supplementary Table 2). It is likely that these chromosomes are particularly sensitive to radiation-induced damage. Most of the genes affected by ^60^Co radiation, regardless of the dosage, appeared to be enriched on chromosomes 11, 17, 19, 16, and 2. Interestingly, chromosome 11 contains the most number of genes whose expression levels were altered by the 1 Gy dosage of radiation, suggesting that this chromosome may be particularly sensitive to ^60^Co radiation. It is possible that these genes located on chromosome 11 are associated with the cell's response to radiation-induced apoptotic signals. We selected these genes for a protein-protein interaction network analysis and categorized the resulting network modules according to their corresponding gene ontologies. The potential relationships among the differentially expressed genes located on chromosome 11 are illustrated in Supplemental Figure 3. Indeed, these genes (FADD, DAK, RARRES3, FUT4, CD44, and CASP1) have functions related to cell death and appear to be involved in cell defense mechanisms.

### 3.3. Disease Associations with DEGs

Even though genes affected by 0.5 Gy of radiation treatment are mostly involved in metabolism, changes in these genes have been associated with various cancers and immune system diseases. It is possible that alterations in basal cellular processes may play an important role in the development of cancer or modulate the risk of cancer. Viral infections are associated with the changes in gene expression across the various radiation dosages tested. This suggests that radiation exposure may weaken the immune defense, increasing the cell's susceptibility to viral attacks (Table 4).

### 3.4. Gene Interaction Network

**Figure 2:**
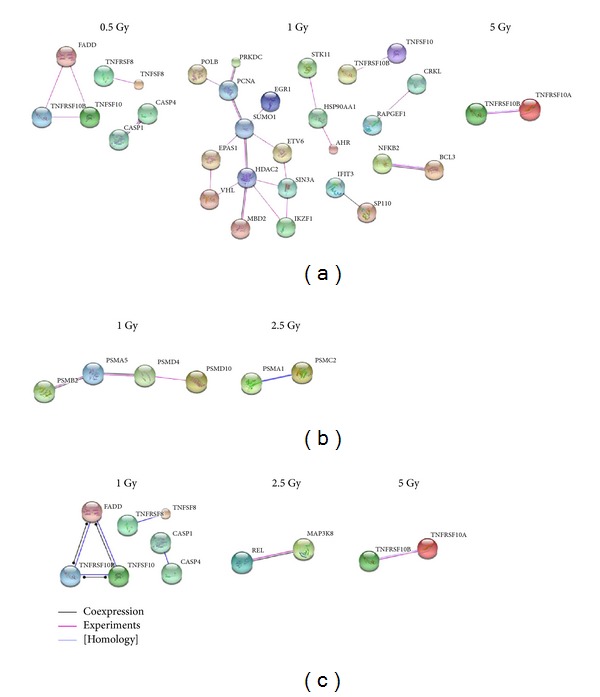
Gene interaction networks specific to the varying doses of ^60^Co radiation exposure. (a) Gene interactions associated with cancer; (b) gene interactions associated with immune system diseases; (c) gene interactions associated with cell death.

The genes and the associated diseases were grouped into broader categories: cancer, immune system diseases, and cell death for visualization of the gene interactions involved in modulating the susceptibility to these cellular abnormalities. This interaction network was constructed based on coexpression and experimental validation.  Figure 2 shows that radiation sensitive genes (FADD, TNFRSF10B, TNFSF10, TNFRSF8, TNFSF8, TNFRSF10A, CASP1, and CASP4) located on chromosome 11 not only are mapped to cancer-, immune system-, and cell death-related diseases but also may be directly interacting with each other, mediating the effects of varying doses of radiation.

## 4. Discussion

The association between low-dose radiation and carcinogenesis remains controversial. In our study, genes responsive to radiation doses (≤1 Gy) were mostly associated with metabolism and signal pathway, whereas radiation doses (>1 Gy) induced expression changes in genes associated with the immune system response and cytokine signaling pathway, as well as cytotoxicity and apoptosis. Although medium-dose radiation could alter the expression of genes involved in metabolic activities, this observation may not be able to establish a direct link between radiation hormesis and cell survival. However, our results suggest that, even at low levels, radiation is able to modulate cellular processes.

At higher dosage levels, the effect of radiation appeared to extend to the immune system and cytokine signaling, inducing changes in cell cycle, proliferation, and cell death, potentially influencing the risk of cancer development in immune cells that are sensitive to radiation [34]. In particular, radiation induces apoptosis in mature T and B lymphocytes responsible for mediating adaptive immunity, causing lethal damage to the precursors of monocytes and granulocytes involved in innate immunity in bone marrow stem cells [35, 36]. In A-bomb survivors, both mature lymphocytes and bone marrow stem cells were severely damaged, weakening the defense of their immune systems against microbial invasion [37, 38]. As a result, many people died from infections. Interestingly, bone marrow stem cells were able to recover from apoptosis induced by infections, suggesting that damage to innate immunity is temporary following radiation exposure. A major subset of T lymphocytes responsible for antigen-specific immunity factors took a long time to recover with diminished CD4 T-cell numbers and function. In compensation for the loss of T cells, the number of B cells is increased in exposed persons [35, 36]. Previous studies also showed decreased number of CD4 T-cells and rising levels of inflammatory proteins in A-bomb survivors' blood samples; the extent of such changes was age-dependent, indicating that radiation exposure may accelerate the aging processes by impairing the immune system. However, as radiation elicits a wide variety of changes in different immune cells, it is difficult to evaluate the effect of radiation on immune system functions. Therefore, there is currently a lack of direct and clear evidence associating the health conditions with persistent abnormalities in the T and B lymphocytes caused by radiation exposure in A-bomb survivors [37, 38]. In our work, we found pathways related immunity were alteration after radiation exposure (Figure 2 and Table 4). The results should provide the putative molecular mechanism involving the harmful effect of radiation exposure to immune-system disruption.

In addition, recent studies have reported that mortality resulting from heart diseases increases with radiation dose in the Life Span Study cohort of the Radiation Effects Research Foundation [39]. Metabolic risk factors, such as hypertension, hypertriglyceridemia, diabetes mellitus, hypercholesterolemia, low high density lipoprotein, and cholesterol, are common complex diseases and are known to increase the risk of coronary heart disease (CHD) [40]. In addition, radiation was found to correlate with visceral fat syndrome, insulin resistance syndrome, and independence of age, gender, or BMI [41]. However, the association between radiation exposure and coronary heart disease (CHD) is unclear. In Tables 2 and 3 shown, metabolic pathway was revealed by enrichment analysis. The results indicate that radiation exposure might enhance the risk to CHD via the metabolic pathway disruption.

We postulate that 1 Gy may be a critical radiation dosage, under which many genes showed altered expression level. Further, at the transcriptome level, the effect of radiation on gene expression may be dosage dependent. Indeed, we observe an increasing number of genes being affected by radiation from 0.5 to 1 Gy dosages. Then, the number of affected genes declined at 2.5 Gy radiation exposure but accelerated again after treatment with 5 Gy of radiation. Although a lot of confounding factors (such as smoking, gender, etc.) have to be considered, epidemiologic evidence is accumulating to suggest that acute exposure to a radiation dosage greater than enhances the risk of circulatory disease for doses higher than 0.5 Gy [42–44]. In line with these observations, we found that, at the 1 Gy radiation dose, not only was the number of altered genes dramatically increasing, but also the number of suppressed genes was greater than the enhanced genes, indicating that cell activity was decreased. The interaction data noted that some genes were associated with cancer, immune system, and cell death, similar to previous studies. These interactions were mapped to different pathways depending on the radiation dosage. Specifically, under 1 Gy of radiation exposure, a lot of interacting genes involved in cancer development, immune system, and cell death responded with changes in expression level. For example, alteration in TNFRSF10B transcript abundance may disrupt cell cycle control and induce the development of carcinogenesis. Changes in genes (PSMA1, 2, 4, 5, and 10) involved in proteasome activity may affect the immune system, diminishing immune defenses and increasing immune system which was affected through proteasome to decrease immune defense and make the cells more susceptible to viral attacks.

From our analysis result in Figure 2, several genes expressions were sensitive to radiation exposure. TNFRSF10B, TNFRSF10A, and TNFRSF8 are members of the TNF-receptor superfamily. TNFRSF10A transduces cell death signal and induces cell apoptosis. In FADD-deficient mice, studies suggested that FADD is required for the apoptosis mediated by TNFRSF10A and TNFRSF10B [45]. TNFRSF8 interacts with TRAF2 and TRAF5 to mediate the activation of NF-*κ*B. TNFRSF8 has been reported to limit the proliferative potential of autoreactive CD8 effector T cells and against autoimmunity and positively regulate apoptosis [46]. High-risk FLT3-ITD mutation of acute myeloid leukemia was associated with high TNFRSF8 expression on myeloblasts [47]. TNFSF10/TRAIL is a cytokine belonging to the TNF ligand family. TNFSF10 induces apoptosis in transformed and tumor cells. TNFSF10 binding to its receptors will trigger the activation of MAPK8/JNK, caspase 8, and caspase 3. TRAIL mediating calcification of aortic valve interstitial cells via the apoptosis mechanism has been reported [48]. FADD is an adaptor molecule and mediates cell apoptotic signal. This protein recruited TNF receptors to initiate the death signaling. CASP1 and CASP4 are members of the caspase family. CASP1 activates the inactive precursor of interleukin-1 involved in inflammasome and induces cell apoptosis in various development stages [49]. CASP4 cleaves and activates its own precursor protein and is required for activation of inflammasome [50]. CASP4 directly activates caspase 9 in endoplasmic reticulum stress-induced neuronal apoptosis [51]. From review of above gene/pathway function, the results indicated carcinogenesis, cell death, and immune system were sensitive to radiation exposure, especially in 1 Gy.

In conclusion, our array-based expression analysis profiled the changes in gene expression in response to varying dosages of radiation exposure. Although our findings require the support from more experimental validations, by integrating publically available tools to perform a series of systematic bioinformatics analyses, we were able to identify a variety of genes involved in metabolism, signaling, immune system, and disease-associated pathways that are sensitive to radiation exposure in a dosage-dependent manner. Follow-up research about the association between these radiation dosages and metabolic mechanism or carcinogenesis would be important to address the effect of low-dose radiation on the development of cancer and metabolic disorders.

## Supplementary Material

Gene ontology enrichment analysis results are given in Table 2 and supplementary table 1. It appears that, starting at a dosage of 0.5 Gy ^60^Co radiation, cells may have already been affected in terms of nucleotide metabolism and signaling pathways responsive to external apoptotic signals.
Beginning at 1 Gy, increasing radiation exposure induces changes in a significant number of genes involved in the immune system processes and programmed cell death pathways. These genes are mostly mapped to chromosomes 2, 11, 16, 17, and 19 (supplementary Table 2). It is likely that these chromosomes are particular sensitive to radiation-induced damages. Most of the genes were affected by 60Co radiation, regardless of the dosage, appeared to be enriched on chromosomes 11, 17, 19, 16, and 2.

## Figures and Tables

**Table 1:** Number of total differentially expressed genes.

Comparison (Gy)	Upregulated genes	Downregulated genes
0 versus 0.5	13	41
0 versus 1	430	398
0 versus 2.5	111	76
0 versus 5	106	299

Standard selection criteria to identify differentially expressed genes are as follows: (1) log_2_⁡|Fold change| ≥ 1 and *P* < 0.05; (2)   log_2_⁡ ratios = “NA” and the differences of intensity between the two samples (duplicated chip) ≥1000 was excluded.

**Table 2:** Top three most enriched biological processes of genes differentially expressed in human peripheral blood mononuclear cells when exposed to varying doses of ^60^Co radiation according to the gene ontology enrichment analysis.

Dose	GO ID	Biological process	*P* value (BH)	Gene symbol
0.5	GO:0045935	Positive regulation of nucleobase-containing compound metabolic process	0.0012^a^	SS18, SMARC2, EPAS1, LHX1, ZNF148, KLF6, F2R
	GO:0051254	Positive regulation of RNA metabolic process	0.0006^a^	TNSF10, MCL1
	GO:2001236	Regulation of extrinsic apoptotic signaling pathway	0.0011^a^	TNSF10, MCL1

1	GO:0002376	Immune system process	0.0018	109 genes^b^
	GO:0006955	Immune response	0.0213	69 genes^b^
	GO:0012501	Programmed cell death	0.03	88 genes^b^

2.5	GO:0044092	Negative regulation of molecular function	0.0299	NKX3-1, DUSP6, CDC27, PRDX3, CAST, PSMC2, BAX, AZIN1, PSMA1, PELI1, CD44, HIPK3, SERPINB2, PI3, SENP2, RLIM, THBS1
	GO:0019221	Cytokine-mediated signaling pathway	0.0299	IL1R1, RANBP2, CCL2, NUP54, CD44, IL1A, IFNGR1, KLF6, EIF4G2, JAK3, CXCR3, HLA-DRB5
	GO:0071345	Cellular response to cytokine stimulus	0.0299	RANBP2, IL1R1, RANBP2, CCL2, NUP54, CD44, IL1A, IFNGR1, KLF6, EIF4G2, JAK3, CXCR3, HLA-DRB5

5	GO:0071310	Cellular response to organic substance	0.009	34 genes^b^
	GO:0019221	Cytokine-mediated signaling pathway	0.0034	USP18, NUP54, IFNAR1, IL1A, CXCR3, EIF4G2, NUP98, TRADD, SOCS3, NUMBL, RANBP2, PML, PTPRN, CD44, JAK3, HLA-DRB5, CAMK2A
	GO:0070887	Cellular response to cytokine stimulus	0.0162	37 genes^b^

^a^The raw *P* value for the GO analysis.

^b^The gene identities are given in the Supplementary Material 1 along with the rest of the GO enrichment analysis results.

**Table 3:** Top three most enriched pathways of genes differentially expressed in human peripheral blood mononuclear cells when exposed to varying doses of ^60^Co radiation according to KEGG pathway enrichment analysis.

Dose (Gy)	KEGG ID	Pathway	*P* value (BH)	Gene symbol
0.5	4610	Complement and coagulation cascades	0.0018	PLAU, F2R

1	1100	Metabolic pathways	2.01*e* − 07	48 genes^a^
	3013	RNA transport	7.28*e* − 05	RANBP2, EIF1AY, EIF4A2, EIF3E, SUMO3, ELAC2, EIF5, EIF1B, NUP98, EIF3H, TACC3, CLNS1A, and SUMO1
	620	Pyruvate metabolism	0.0001	ACAT1, MDH1, ACACB, PKM, DLD, HAGHL, and ACACA

2.5	4010	MAPK signaling pathway	2.15*e* − 06	1L1R1, ELK4, MDUSP6, PLA2G5, MAP4K4, MAP3K8, MAP4K3, PPM1A, IL1A, and PRKACB
	4210	Apoptosis	0.0034	1L1R1, 1L1A, PRKACB, and BAX
	4640	Hematopoietic cell lineage	0.0034	1L1R1, CD44, IL1A, and HLA-DRB5

5	4120	Ubiquitin mediated proteolysis	0.0007	ANAPC1, PARK2, CDC27, PML, SKP1, SAE1, SYVN1, and SOCS3
	4650	Natural killer cell mediated cytotoxicity	0.0034	PIK3R3, TNFRSF10, NRAS, PRKCG, IFNAR1, and TNFRSF10
	4080	Neuroactive ligand-receptor interaction	0.0065	HTR7, SSTR5, GABRB2, TAAR9, S1PR2, GHRHR, TAAR5, CCKAR, and VIPR2

^a^The gene identities are given in the Supplementary Material 2 along with the rest of the KEGG pathway enrichment analysis results.

**Table 4:** Top 10 diseases overrepresented by the differentially expressed genes under the exposure of varying doses of  ^60^Co exposure in human PBMC.

0.5 Gy	1 Gy	2.5 Gy	5 Gy
Glioma	HIV	Necrosis	Neuroblastoma
Carcinoma, small cell	Leukemia	Hernia	Hernia
Neoplasms	Viral diseases	Viral diseases	Hematologic
Cancer or viral infections	Cancer or viral infections	HIV	Peripheral
Carcinoma, hepatocellular	Shock	Immunologic deficiency syndromes	Necrosis
Liver neoplasms	Immunologic deficiency syndromes	Chorioamnionitis	Therapy-related acute myeloid leukemia (t-ML)
Carcinoma	Death	Retroviridae infections	Connective tissue diseases
Hypercortisolism	Sexually transmitted diseases	Preterm rupture of membranes	Autoimmune diseases
Glioblastoma	*Lentivirus* infections	*Lentivirus* infections	Disease susceptibility
Immune system diseases	Retroviridae infections	Periodontitis	Genetic predisposition to disease

All disease associations were filtered by BH-adjusted *P* value <0.05.
