# Supplementary material for: Gene Expression Profiling of Biological Pathway Alterations by Radiation Exposure
Source: Biomed Res Int. 2014 Sep 8;2014:834087. doi: 10.1155/2014/834087 (PMC4170887; doi:10.1155/2014/834087)

Supplement figure 1. PCA plot

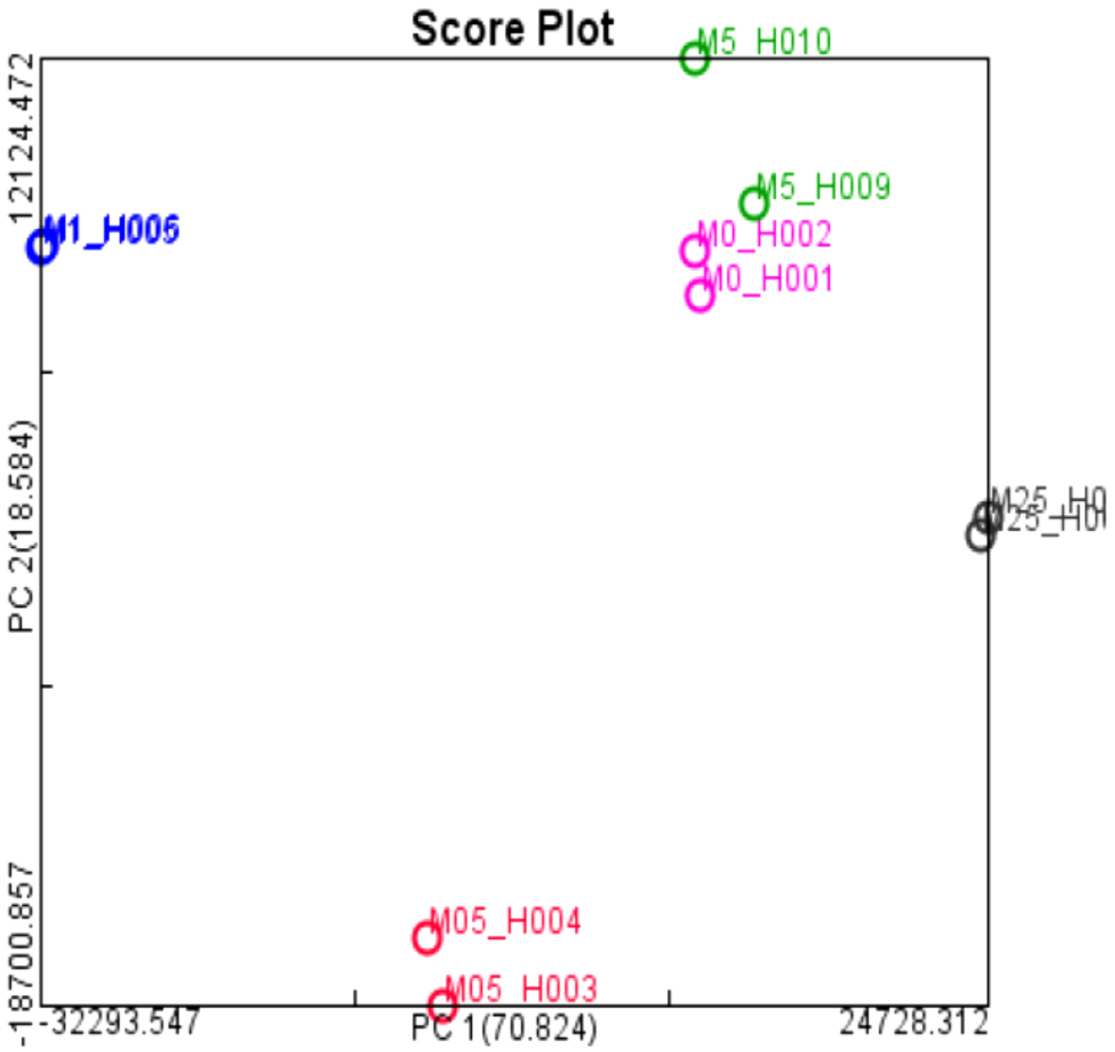

The variable of the first three principal components (PC1, PC2, PC3) for this study are 70.82%, 18.58% and 7.69% respectively. A subset of differential genes was selected for PCA analysis. An intensity filter was used to select genes where the difference between the maximum and minimum intensity values exceeds 400 among all microarrays. For this microarray project, the number of genes clustered was 238.

Supplement figure 2. Clustering analysis

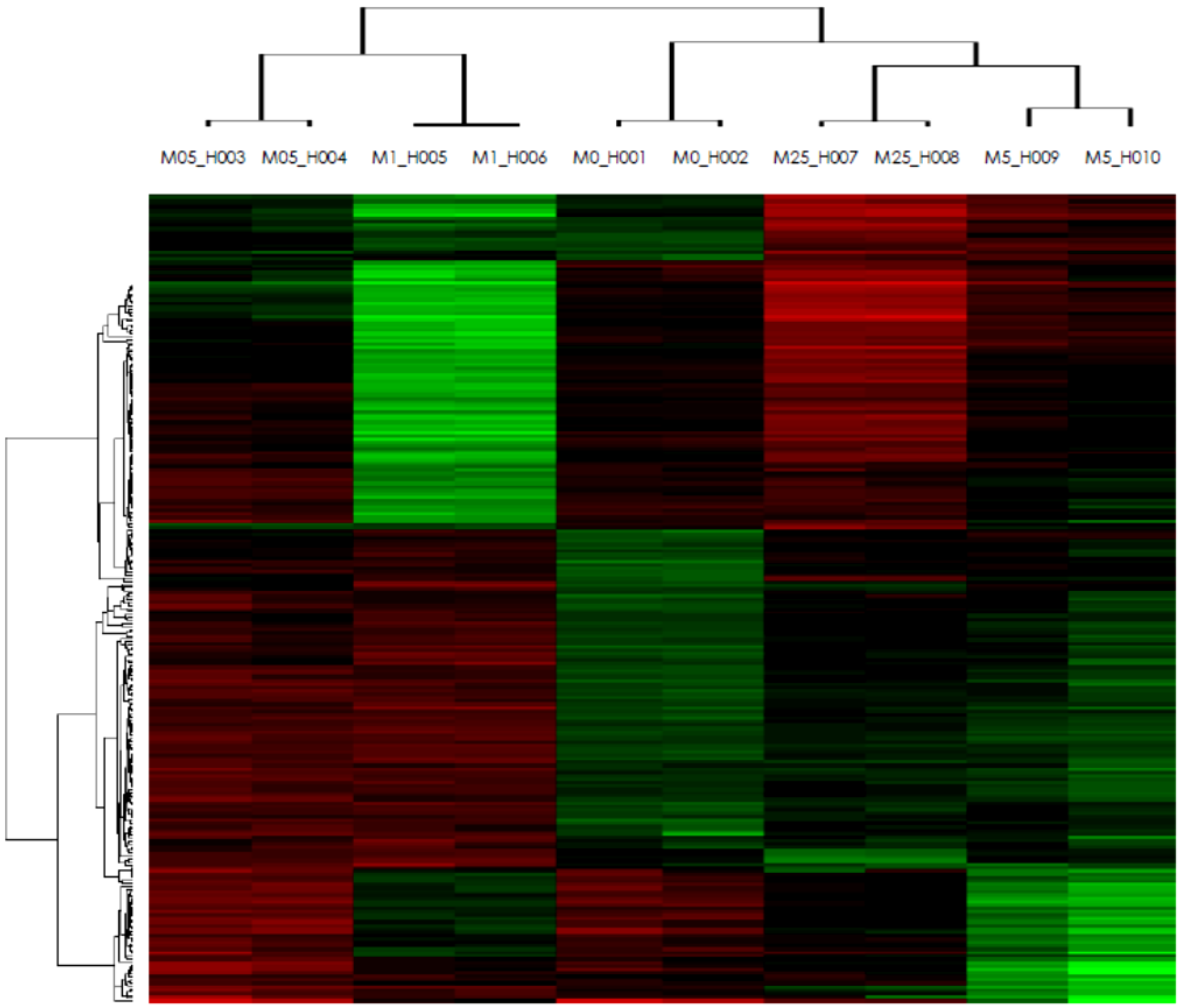

Clustering was performed to visualize the correlations among the replicates and varying sample conditions. Up- and down-regulated genes are represented in red and green colors, respectively. A subset of differential genes was selected for clustering analysis. An intensity filter was used to select genes where the difference between the maximum and minimum intensity values exceeds 400 among all microarrays. For this microarray project, the number of genes clustered was 238.

Supplementary Table 1.

KEGG pathway	ID	C	O	E	R	rawP	adjP
Metabolic pathways	ID:01100	1130	48	18.16	2.64	1.55E-09	2.01E-07
gene_symbol	ACACB	DNMT3A	SUCLG1	POLR1A	DGKA	DAK	OAT
	PMM2	GLUL	ST3GAL4	LTC4S	BDH1	PPCDC	CTPS1
	PTDSS2	AHCY	PCYT2	SPTLC1	TALDO1	DLD	ATP6V1E1
	POLR1E	GLYCTK	ACACA	ATP6V1G1	SMS	GAPDH	NDST2
	C5orf4	PNP	SAT1	AMD1	IL4I1	SCP2	OGDH
	PKM	FUT4	NDST1	HPRT1	ATP5G3	KYNU	COASY
	IMPDH1	ACAT1	MDH1	HMBS	DHCR7	BTD	

C: the number of reference genes in the category

O: the number of genes in the gene set and also in the category

E: the expected number in the category

R: ratio of enrichment

rawP: p value from hypergeometric test

adjP: p value adjusted by the multiple test adjustment

Supplementary Table 2. Chromosomes containing the most number of differentially expressed genes categorized according to the varying doses of ^{60}Co radiation exposure.

Chromosome	0.5 Gy	1 Gy	2.5 Gy	5 Gy
11	None	AMICA1, MPEG1, DAK, LOC100128242, PPP2R5B, ETS1, TMEM179B, FAM111A, APLP2, ESAM, ST3GAL4, TMPRSS5, CASP1, RPS6KB2, VPS11, SIK3, OAF, DRAP1, THYN1, VPS37C, FADD, FUT4, CCDC86, MALAT1, LGALS12, CCDC85B, RARRES3, OVOL1, ACAT1, HMBS, ESRRA, DHCR7	PSMA1,SPTY2D1 ,CD44, OR52M1, EIF4G2, HIPK3, NRIP3	None
17	EVI2B[a], CCDC47, LHX1	EVI2B[a], MIF4GD, GGNBP2, CSHL1, CD79B, SDF2, AZI1, SKAP1, GOSR1, HDAC5, ITGA2B, SPAG5, USP36, RPTOR, LRRC45, GALR2, MBTD1, ACBD4, SLC25A19, PLEKHM1, TUBG1, SLC38A10, CYGB, PCYT2, OSBPL7, RNF157, NKIRAS2, CSNK1D, ACACA	No gene	None
19	None	BRD4, PRKCSH, C19orf25, ILF3, MAP1S, GIPC1, ABHD8, ZNF44, RANBP3, SH3GL1, HOMER3, HAUS8, ZNF555, IZUMO4, LMNB2, NACC1, YIPF2, BST2, CCDC94, LPHN1, TNPO2, STK11, ILVBL, NR2F6, NMRK2,LONP1, GPX4, TLE2	None	None

16	None	UNKL, ITGAL, NUDT16L1, NPRL3 , DECR2, CRYM, C16orf72, ZNF668, SEC14L5, IGSF6, PMM2, RAB40C, YPEL3, HBQ1, SNRNP25, ANKS3, ABCC6, HAGHL, PTX4, METRN, MLST8	None	None
2	None	None	IL1R1, RANBP2[a]	NEU4, RANBP2[a], CYTIP, MCM6, CCNYL1, CCL20, CYP20A1, C2orf72, AGFG1, IL1A, ICOS, NFE2L2, RNF103, TCF7L1

[a]indicates that the gene is up-regulated under the exposure of two or more radiation dosage.

Supplement figure 3 protein-protein interaction network analysis. Genes expression associated with the cell’s response to radiation-induced signaling pathway according to their corresponding gene ontologies.

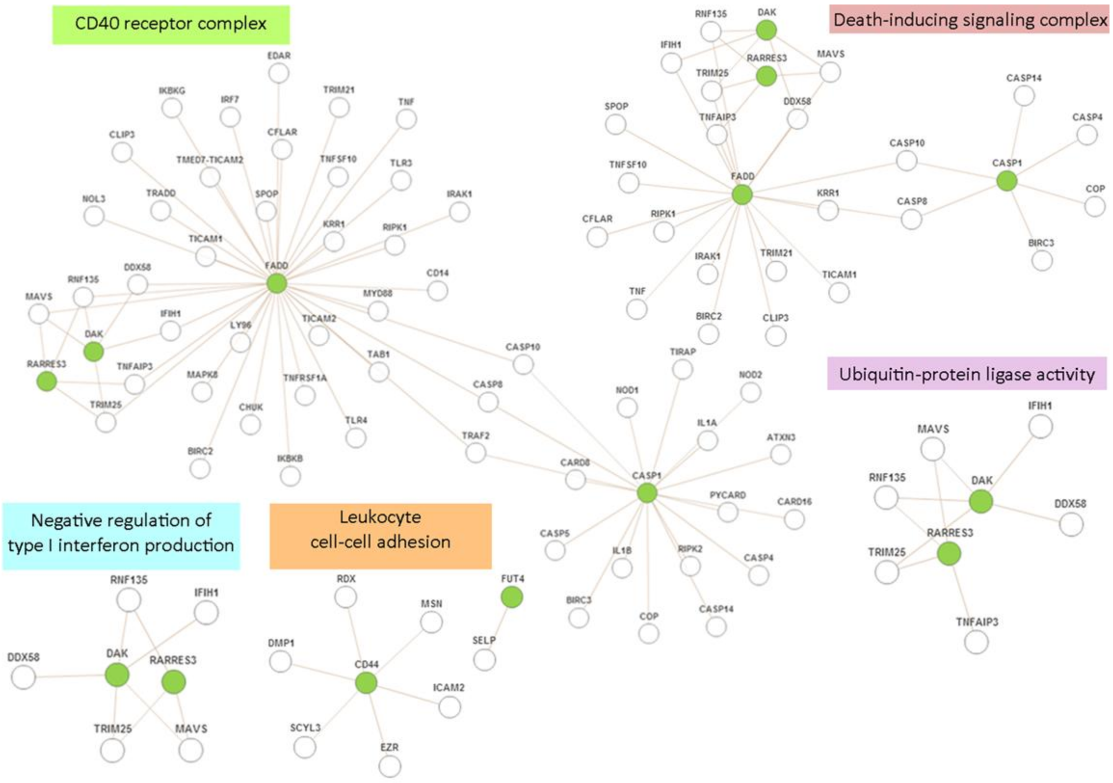

Supplement: Supplementary file 1 — Gene ontology enrichment analysis results are given in Table 2 and supplementary table 1. It appears that, starting at a dosage of 0.5 Gy 60Co radiation, cells may have already been affected in terms of nucleotide metabolism and signaling pathways responsive to external apoptotic signals. Beginning at 1 Gy, increasing radiation exposure induces changes in a significant number of genes involved in the immune system processes and programmed cell death pathways. These genes are mostly mapped to chromosomes 2, 11, 16, 17, and 19 (supplementary Table 2). It is likely that these chromosomes are particular sensitive to radiation-induced damages. Most of the genes were affected by 60Co radiation, regardless of the dosage, appeared to be enriched on chromosomes 11, 17, 19, 16, and 2. [file 834087.f1.pdf]
